# Physiology to the Pleiotropic Role of RNAs: Prospecting Novel Therapies

**DOI:** 10.1155/2014/735374

**Published:** 2014-07-01

**Authors:** Maria Chiara Maiuri, Daniela De Stefano, Ammad Ahmad Farooqi

**Affiliations:** ^1^INSERM, U848, Institut Gustave Roussy, PR1, 39 rue Camille Desmoulins, Villejuif, 94805 Paris, France; ^2^Dipartimento di Farmacia, Università Degli Studi di Napoli Federico II, Via D. Montesano 49, 80135 Napoli, Italy; ^3^Laboratory for Translational Oncology and Personalized Medicine, Rashid Latif Medical College, 35 Km Ferozepur Road, 54600 Lahore, Pakistan

The purpose of this special issue is to point the attention to the RNA functions, which in the last decades emerged as extremely important. Particularly, physiopathological roles of microRNA, short hairpin RNA, and small interfering RNA have been reported, in turn leading to the opportunity to consider RNAs as both novel therapeutic targets and tools. The contributions of this special issue cover a variety of subjects and reflect the complexity of the field.

In particular, RNA is a difficult molecule, due to its rapid degradation. Several strategies have been described to overcome these issues, in order to render the use of RNA possible. M. Gaglione et al. reported chemical modifications of siRNAs containing terminal amide linkages by introducing hydroxyethylglycine PNA (hegPNA) moieties at 5′, at 3′ positions, and on both terminals and demonstrated that some of these modifications are compatible with the RNAi machinery, as they markedly increased the resistance to serum-derived nucleases. Indeed, Z. Deng et al. reported the role of PNPase as a major enzyme of sRNAs degradation.

I. Scognamiglio et al. describe the development and effects of stable nucleic acid lipid vesicles (SNALPs) encapsulating miR-34a to treat multiple myeloma. In this study, the authors show that it is possible to overcome the targeted delivery of SNALPs as well as miRNA encapsulation efficiency drawbacks by conjugating SNALPs with the transferrin and chemically modifying the miRNA with a 2′-O-methylation. The formulation was effective in reducing tumor growth.

The miRNAs are the subject of another contribution. A. Hsu et al. demonstrated that an alteration of miRNA levels may be used as diagnostic markers of myocardial infarction. The differentially expressed miRNAs were evaluated in a separate cohort of 62 subjects to show that serum miR-486-3p and miR-150-3p were upregulated while miR-126-3p, 14 miR-26a-5p, and miR-191-5p were significantly downregulated suggesting that serum miRNAs may be used as potential diagnostic biomarkers. F. Amato et al. reported the effectiveness of an inhibitor of miR-5093p for the regulation of cystic fibrosis-related gene expression.

Primary miRNA (pri-miRNA) is the subject of M. B. Mowa and colleagues study. The authors investigated the utility of the murine transthyretin receptor (MTTR) promoter for expression of artificial anti-HBV primary miRNA (pri-miRNA) sequences to develop their use in liver-specific transcription regulatory elements. They demonstrated that the expression of anti-HBV pri-miR mimics from MTTR promoter is well suited to countering HBV replication and development of HD Ads through attenuation of their immunostimulatory effects.

The present issue constitutes an important update in a constantly developing field. The efforts to carry on these studies will, possibly, generate new therapeutic opportunities in the near future.

*Maria Chiara Maiuri* *Daniela De Stefano* *Ammad Ahmad Farooqi*

